# The Distinguishing Bacterial Features From Active and Remission Stages of Ulcerative Colitis Revealed by Paired Fecal Metagenomes

**DOI:** 10.3389/fmicb.2022.883495

**Published:** 2022-06-21

**Authors:** Ran Zhu, Junrui Tang, Chengfeng Xing, Qiong Nan, Guili Liang, Juan Luo, Jiao Zhou, Yinglei Miao, Yu Cao, Shaoxing Dai, Danfeng Lan

**Affiliations:** ^1^State Key Laboratory of Primate Biomedical Research, Institute of Primate Translational Medicine, Kunming University of Science and Technology, Kunming, China; ^2^Department of Gastroenterology, The First Affiliated Hospital of Kunming Medical University, Yunnan Province Clinical Research Center for Digestive Diseases, Kunming, China; ^3^Department of Cardiac Surgery, The First People’s Hospital of Yunnan Province, Kunming, China

**Keywords:** ulcerative colitis, metagenomics analysis, paired samples, intestinal microbiota, biomarker panel

## Abstract

Ulcerative colitis (UC) is a serious chronic intestinal inflammatory disease, with an increased incidence in recent years. The intestinal microbiota plays a key role in the pathogenesis of UC. However, there is no unified conclusion on how the intestinal microbiota changes. Most studies focus on the change between UC patients and healthy individuals, rather than the active and remission stage of the same patient. To minimize the influences of genetic differences, environmental and dietary factors, we studied the intestinal microbiota of paired fecal samples from 42 UC patients at the active and remission stages. We identified 175 species of microbes from 11 phyla and found no difference of the alpha and beta diversities between the active and remission stages. Paired *t*-test analysis revealed differential microbiota at levels of the phyla, class, order, family, genus, and species, including 13 species with differential abundance. For example, *CAG-269 sp001916005*, *Eubacterium F sp003491505*, *Lachnospira sp000436475*, et al. were downregulated in the remission, while the species of *Parabacteroides distasonis*, *Prevotellamassilia sp900540885*, *CAG-495 sp001917125*, et al. were upregulated in the remission. The 13 species can effectively distinguish the active and remission stages. Functional analysis showed that the sporulation and biosynthesis were downregulated, and the hydrogen peroxide catabolic process was upregulated in remission of UC. Our study suggests that the 13 species together may serve as a biomarker panel contributing to identify the active and remission stages of UC, which provides a valuable reference for the treatment of UC patients by FMT or other therapeutic methods.

## Introduction

Inflammatory bowel disease (IBD) is a group of chronic non-specific intestinal inflammatory diseases including ulcerative colitis (UC) and Crohn’s disease (CD) mainly (Kobayashi et al., 2020). The incidence of UC, which is thought to primarily occur in European and American countries, is increasing in developing countries year by year since the 21st century (Ng et al., 2017). The main clinical symptoms are abdominal pain, diarrhea, and hematochezia, the relapses and remissions of disease alternate repeatedly, which impacts the life quality of UC patients and causes a serious medical burden (Ordás et al., 2012). Alterations in diversity and composition of the intestinal microbiota, termed dysbiosis, play an important role in the pathogenesis of UC (Ungaro et al., 2017). Although the treatment outcome has improved with the use of biologic agents such as infliximab, a certain proportion of UC patients are resistant to pharmacological therapy and have to undergo colectomy (Ordás et al., 2012). With a series of basic studies, many new treatment strategies for IBD have emerged, such as fecal microbiota transplantation (FMT; Moayyedi et al., 2015).

The emergence of high-throughput amplicon sequencing opened a new field of research, with potential implications for diagnosing UC. Characterization of the fecal microbiome has been the focal point for most UC microbiome studies, as fecal samples are noninvasive and easy to collect (Lin et al., 2021). Metagenomics, a new approach to studying microbiota, can dispense with the process of isolating and culturing individual microbes, and study part or all of intestinal microbiota directly (Zengler and Palsson, 2012). At present, most studies focus on the change of intestinal microbiota between UC patients and healthy individuals. It is reported that patients with IBD may harbor reduced bacterial diversity and lower abundance of Firmicutes vs. Proteobacteria compared with healthy people (Goethel et al., 2018). However, the intestinal microbiota is affected by many factors, including heredity, environmental influences, dietary factors, and so on (Goodrich et al., 2014; Singh et al., 2017; Rothschild et al., 2018). There are few studies on how the intestinal microbiota changes in the active and remission stages of UC patients, and no consensus has been reached so far.

To minimize the influences of these interference factors, we focused on the same patients of UC at different stages. The specific microbiota was identified from the paired fecal samples using metagenomic sequencing, paired *t*-test analysis, and functional annotation in this study. Our studies aimed to determine the changes of intestinal microbiota in the active and remission stages of UC and provide a valuable clinical reference for the treatment of UC patients by FMT or other therapeutic methods.

## Materials and Methods

### Study Population

A total of 42 UC patients were recruited at the First Affiliated Hospital of Kunming Medical University from 2019 to 2020, including 22 men and 20 women. The diagnosis of UC was based on clinical, radiological, endoscopic, and histopathological findings, in accordance with the Third European Evidence-based Consensus on Diagnosis and Management of Ulcerative Colitis (Harbord et al., 2017). Those patients with mental illness and the severe damage of heart, liver, lung, or kidney functions were excluded. The 42 fecal samples of the active stage were collected from the UC patients who were hospitalized in the gastroenterology department, while the fecal samples of the remission stage were collected when they attended the health lectures for IBD patients. They had no symptoms of abdominal pain, diarrhea, fever, and hematochezia at the remission. The time interval between remission and active sample collection was approximately 3 months. UC disease activity evaluation was performed according to the modified Mayo scoring system. The UC patients were classified into four disease activities as follows: (1) remission (0 ≤ Mayo score ≤ 2); (2) mildly active disease (3 ≤ Mayo score ≤ 5); (3) moderately active disease (6 ≤ Mayo score ≤ 10); and (4) severely active disease (11 ≤ Mayo score ≤ 12; D’Haens et al., 2007).

### Ethical Statement

This study was approved by the Institutional Review Board for Clinical Research of the First Affiliated Hospital of Kunming Medical University. Informed consent was obtained from all subjects.

### Metagenomic Sequencing

Fecal samples from UC patients were collected in centrifuge tubes and stored at −80°C until DNA extraction. DNA was extracted from fecal samples according to the operating procedures of the genomic DNA kit (Novogene, Beijing, China). Sequencing libraries were generated using the NEB Next Ultra DNA Library Prep Kit for Illumina® (New England Biolabs, Ipswich, Massachusetts, United States). The quality of sequencing libraries was assessed using an Agilent Bioanalyzer 2100 system. The library was sequenced using 150 bp paired-end sequencing on an Illumina Hiseq 4000 platform.

### Metagenome Assembly and Annotation

Metagenomic reads were processed using the ATLAS pipeline (2.4.1; Kieser et al., 2020), which performs quality control, assembly, annotation, binning, and read mapping. Quality control was performed with the BBmap suite (v38.86; Bushnell, 2022) tools to eliminate adapters, remove PCR duplicates, and then trim and filter reads based on quality scores and lengths of reads. Contaminations from the human genome were filtered out by KneadData (v0.7.2). Paired-end reads were assembled using the metaSPAdes (v3.12.0; Nurk et al., 2017) assembly tool. Prodigal (v2.6.3; Hyatt et al., 2010) was used to predict n reading frames (ORFs) in contigs and translated gene products were mapped to the eggnog catalog using DIAMOND (v2.0.2; Buchfink et al., 2021). Contigs were then binned using MetaBAT2 (v2.14; Kang et al., 2019) and MaxBin2 (v2.2.4; Wu et al., 2016), followed by DASTool (v1.1.2–1; Sieber et al., 2018) as a final binner to dereplicate, aggregate, and score the bins and create high-quality metagenome assembled genomes (MAGs), which had at least 50% completeness and <10% contamination based on the estimation by CheckM (v1.1.2; Parks et al., 2015) were clustered (95% average nucleotide identity) resulting in 188 representative genomes (referred later as genomes). Because some of the same genomes were identified in multiple samples, resulting in multiple bins for the same MAG, dRep (v2.2.2; Olm et al., 2017) was also used to select the best bin for each MAG. To assess the contribution of the constructed MAGs to the functional potential of the gut microbiome, the predicted gene and proteins extracted by Prodigal during the CheckM pipeline were compared to the EggNOG database 5.0 using eggnog-mapper (v2.1.4; Cantalapiedra et al., 2021). From this output, Kyoto Encyclopedia of Genes and Genomes (KEGG) annotation, COG (Clusters of Orthologous Groups of proteins) annotation, and GO (GeneOntology) annotation were extracted.

### Diversity Analysis, Abundance Analysis, and Enrichment Analysis

Microbiome data is compositional because the information that abundance tables contain is relative. In a microbiome abundance table, total count constraint induces strong dependencies among the abundances of the different taxa. The total number of counts does not exceed the specified sequencing depth. The observed raw abundances and the total number of reads per sample are non-informative. We used CODA methods to analyze microbiome data, which were recommended by researchers (Gloor et al., 2017; Rivera-Pinto et al., 2018; Calle, 2019). The R function “plot_anova_diversity” in the package microbiomeSeq (v0.1) was used for statistical analysis of alpha-diversity. Principal component analysis (PCA) of CLR-transformed (centric log2 ratio) species abundance of microbiota was conducted with the “prcomp” function in the package stats (v4.0.2). The abundance of each genome was quantified across samples by mapping the reads to the non-redundant MAGs and determining the median coverage across each genome. For the relative abundance, we take the coverage over the genome, not the raw counts. This implicitly normalizes for genome size. The coverage is calculated as the median of the coverage values calculated in 1 kb blocks. Differential abundance tests were conducted with the ALDEx2 v1.20.0 Bioconductor package. The R Biocondutor packages clusterProfiler (v3.16.0) was used for functional enrichment analysis over differential microbiota sets. Whereas the visualization of enrichment analysis was performed with the R Bioconductor packages ggplot2 (v3.3.5). The partial least square (PLS) analysis was performed through the “plsr” function (pls R package v2.8-0).

## Results

### Quality Control and Data Processing

**Figure 1:**
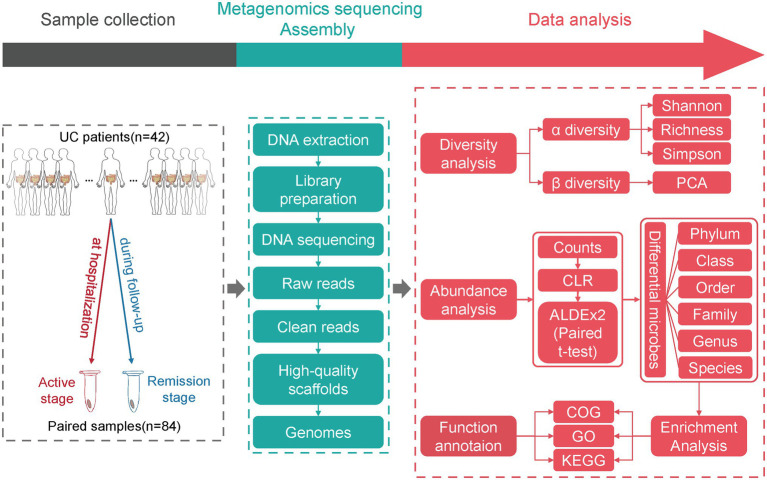
The study design and flow chart of data analysis. The fecal samples from UC patients in active and remission stages were collected. After DNA extraction from fecal samples, library preparation, and metagenomic sequencing, the raw data were subjected to a series of analyses.

The clinical parameters of UC patients were shown in Table 1. After DNA extraction from fecal samples, library preparation and metagenomic sequencing, the raw data was subjected to a series of analyses (Figure 1). Seven samples were removed due to the high host contamination of these samples. A total of 35 samples were retained for further analysis (Supplementary Table 1). The filtration process removed *homo sapiens* sequences and low-quality reads, leaving over 1 million clean reads of each sample (Supplementary Figure 1; Supplementary Table 2). The age of male (*n* = 20) participants ranged from 16 to 68 (Mean age = 45.50, SD = 12.75), and the age of female (*n* = 15) participants ranged from 21 to 65 (Mean age = 41.93, SD = 12.83). There was no significant bias for the sample distribution in the extent of lesion, disease severity, medications and so on (Table 1).

**Table 1:** Clinical parameters of patients with UC.

Parameters	Pre-filtration (*n* = 42)		Post-filtration (*n* = 35)	
Gender	Male:22	Female:20	Male:20	Female:15
Min–max age	16–68	21–65	16–68	21–65
Mean age ± SD	44.45 ± 13.53	43.10 ± 13.16	45.50 ± 12.75	41.93 ± 12.83
Nationality (Han nationality%)	100	90	100	87
Residence (town%)	55	65	55	53
**Clinical type**^①^				
F	5(22.7%)	1(5.0%)	5(25%)	1(6.7%)
C	17(77.3%)	19(95.0%)	15(75%)	14(93.3%)
**Extent of lesion**^②^				
E1 (Proctitis)	4(18.2%)	3(15.0%)	4(20.0%)	3(20.0%)
E2 (Left sided colitis)	7(31.8%)	6(30.0%)	6(30.0%)	5(33.3%)
E3 (Pancolitis)	11(50.0%)	11(55.0%)	10(50.0%)	7(46.7%)
**Disease severity**^③^				
Mild	5(22.7%)	3(15.0%)	5(25.0%)	3(20.0%)
Moderate	8(36.4%)	7(35.0%)	7(35.0%)	7(46.7%)
Severe	9(40.9%)	10(50.0%)	8(40.0%)	5(33.3%)
**Medications**^④^				
5-ASA	11(50%)	7(35.0%)	11(55.0%)	7(46.7%)
Immunosuppressants	1(4.5%)	2(10.0%)	1(5.0%)	2(13.3%)
Anti-TNF-α agents	0	1(5.0%)	0	1(6.7%)
Combination therapy	10(45.5%)	10(50.0%)	8(40.0%)	5(33.3%)
**Interval between episode and the last**^⑤^				
Less than 12 months	5(22.7%)	2(10%)	5(25%)	2(13.3%)
More than 12 months	17(77.3%)	18(90%)	15(75%)	13(86.7%)
**Duration of medication (days)**^⑥^	10.32 ± 3.78	10.70 ± 3.59	9.95 ± 3.78	9.93 ± 3.73

① Clinical type: F (first episode: the first episode without previous history), C (chronic relapsing type: symptoms reappear after remission).

② Extent of lesion: the extent of lesion is classified according to the Montreal typing.

③ Disease severity: The severity of disease is based on the modified mayo score.

④ *Medications: 5-aminosalisylic acid (5-ASA): mesalazine; Immunosuppressants: azathioprine, 6-mercaptopurine, thalidomide; Anti-TNF-α agents: Infliximab; Combination therapy: corticosteroids +5-ASA/ immunosuppressants, Anti-TNF-α agents+5-ASA/ immunosuppressants.*

⑤ Interval between episode and the last: the time interval between this episode and the last.

⑥ Duration of medication: duration of treatment from admission to remission.

### The Alpha and Beta Diversities Showed No Difference Between the Two Stages

**Figure 2:**
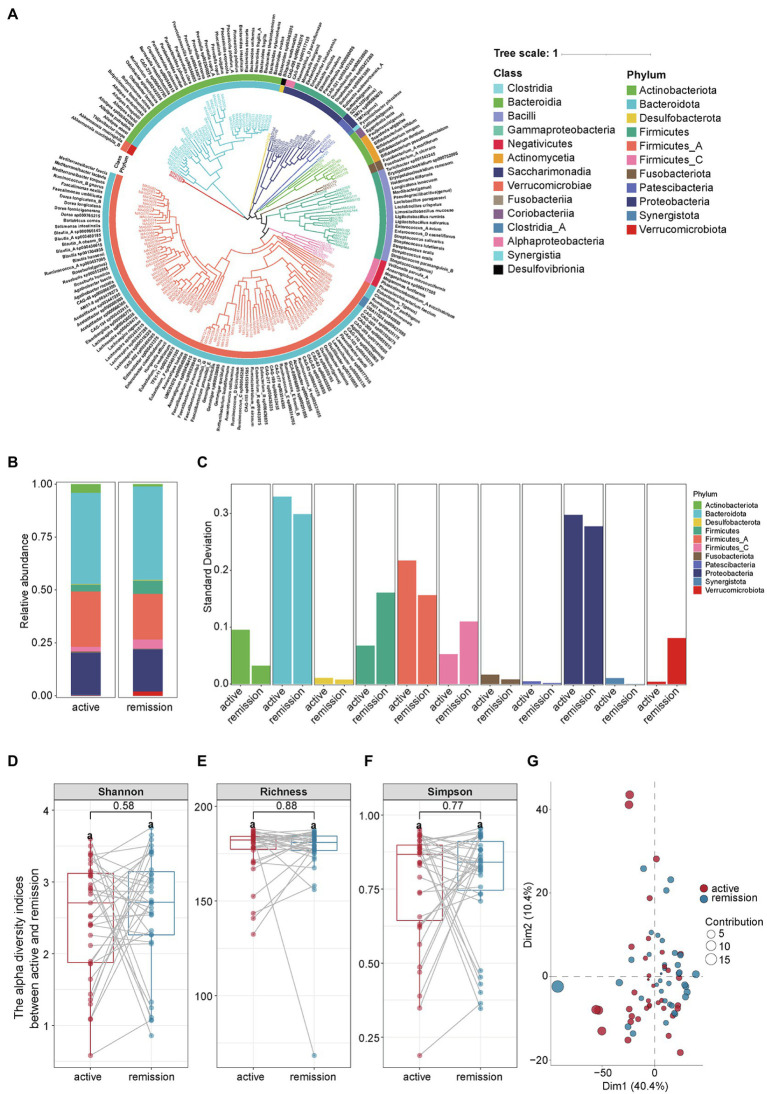
The composition and diversity of intestinal microbiota in fecal samples from UC patients. **(A)** The composition of intestinal microbiota in the active and remission stages of UC. **(B)** The abundance of intestinal microbiota at the phyla level in the active and remission stages of UC. **(C)** Comparison of the abundance variance of different phyla in the active and remission stages. **(D–F)** The paired *t*-test of alpha diversity indicators including the Shannon index, Richness index and Simpson index. Statistical significance was defined as *p* < 0.05. **(G)** Communities cluster using Principal Component Analysis (PCA). Dim1 and Dim2 are plotted on x- and y-axes. Each point corresponds to each sample. The percentage of variation analyzed by the plotted principal components is shown on the axes. Red and blue indicate the active and remission stage of UC, respectively.

From 35 paired fecal samples, we identified 175 species of microbes that belong to 11 phyla, 15 classes, 25 orders, 43 families, and 118 genera (Figure 2A). At the phylum level, the main compositions of intestinal microbiota in the remission and active stages were Bacteroidota (44.21% vs. 43.13%), Firmicutes A (21.66% vs. 26.23%), Proteobacteria (19.91% vs. 19.85%), Actinobacteriota (1.17% vs. 4.16%), and Firmicutes (6.20% vs. 3.19%), Firmicutes C (4.36% vs. 2.19%; Figure 2B). The relative abundance of each phylum fluctuated between the active and remission stages in varying degrees (Figure 2C). The detailed proportion of phyla in each sample was shown in Supplementary Figure 2. Then, we compared the alpha and beta diversities between the samples the active and remission stages. Alpha diversity reflected the abundance and uniformity of microbial species in a population, including the Shannon index, richness index, and Simpson index. The three indexes had no significant differences between the two stages (Figures 2D–F). The values of *p* for the Shannon index, richness index, and Simpson index are 0.58, 0.88, and 0.77, respectively. Beta diversity reflected the diversity of microbial species between samples. Principal component analysis (PCA) showed a great overlap of microbial species between the samples of active and remission stages of UC effectively (Figure 2G).

### Paired *t*-Test Identified 13 Species With Differential Abundance

**Figure 3:**
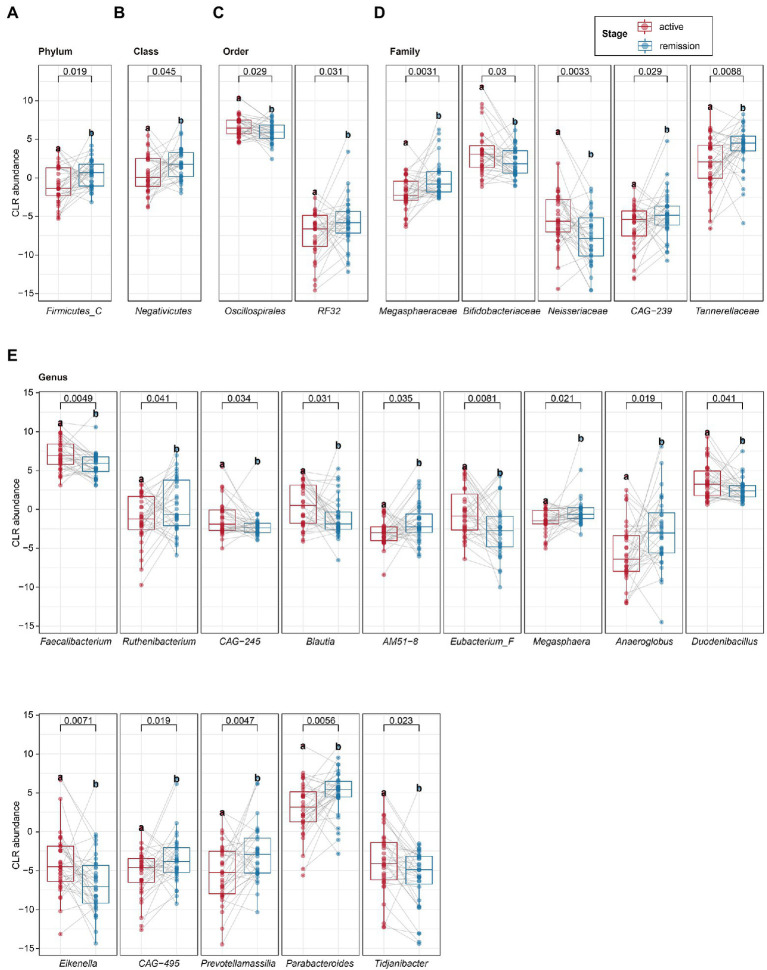
The paired *t*-test analysis of intestinal microbiota. **(A–E)** The different microbiota at the phylum, class, order, family, and genus levels was identified by the paired *t*-test. Statistical significance was defined as *p* < 0.05. *p < 0.05, **p < 0.01, and ***p < 0.001.

To determine the differential microbiota composition between the active and remission stages of UC patients, the paired *t*-test was applied to analyze microbiota composition at different levels of the phyla, class, order, family, genus, and species. The relative abundance of Firmicutes C (at the phylum level) and Negativicutes (at the class level) was upregulated in the remission stage (Figures 3A,B). The relative abundance of RF32 and Oscillospirales (at the order level) was upregulated and downregulated in the remission stage, respectively (Figure 3C). The relative abundance of Tannerellaceae, Megasphaeraceae, and CAG-239 (at the family level) was upregulated, while that of Bifidobacteriaceae and Neisseriaceae (at the family level) was downregulated in the remission stage (Figure 3D). At the genus level, we identified 14 genera with differential abundance (Figure 3E). The relative abundance of seven genera (*Faecalibacterium*, *CAG*–*245*, *Blautia*, *Eubacterium F*, *Duodenibacillus*, *Eikenella*, and *Tidjanibacter*) was upregulated in the remission stage. In contrast, the relative abundance of the other genera (*Ruthenibacterium*, *AM51–8*, *Megasphaera*, *Anaeroglobus*, *Prevotellamassilia*, *CAG*–*495*, and *Parabacteroides*) was downregulated in the remission stage. Furthermore, at the species level, we identified 13 species with differential abundance, of which the relative abundance of five species was upregulated, and that of eight species was downregulated in the remission stage (Table 2). Linear discriminant analysis Effect Size (LEfSe) analysis, one classic method to find out the differential microbiota, was also used. LEfSe revealed that the relative abundance of Firmicutes C, Negativicutes, Megasphaeraceae, Tannerellaceae, *Anaeroglobus*, *Parabacterioides*, *AM51 8*, and *Prevotellamassilia* increased significantly in the remission stage, while the relative abundance of Neisseriaceae, *Faecalibacterium*, *Blautia*, *Eubacterium F*, *Duodenibacillus*, and *Eikenella* increased significantly in the active stage (Supplementary Figure 3A). The differential microbiota found by paired *t*-test not only contained all found by LEfSe analysis but also found new microbiota such as Oscillospirales, *Ruthenibacterium* and so on. Then, we conducted partial least square (PLS) analysis to evaluate the effect of other covariates. We quantified the possible effects of the covariates (including age, gender, the extent of lesion, severity, stage, and so on) on the abundance of 13 selected species (Supplementary Figure 3B). The stage and severity of UC were the main factors for the abundance, and the covariates (age, gender, the extent of lesion, and so on) had basically no effect on the abundance of the 13 selected species.

**Table 2:** The differential microbiota at the species level identified by paired *t*-test.

Species	Median clr value		Diff.btw	wePvalue	Change
	Active	Remission			
Parabacteroides distasonis^***^	0.861031	4.872401	3.124384	0.000222	UP
CAG-269 sp001916005^**^	−3.419122	−6.139985	−2.865523	0.002689	DOWN
Eubacterium_F sp003491505^**^	−0.632760	−2.346559	−2.157945	0.005360	DOWN
Lachnospira sp000436475^**^	1.998101	0.797277	−1.432573	0.005422	DOWN
Prevotellamassilia sp900540885^**^	−5.243744	−2.852779	2.148754	0.007819	UP
Eikenella corrodens^*^	−4.090419	−6.880818	−2.678801	0.013392	DOWN
CAG-495 sp001917125^*^	−7.240989	−5.428500	2.121978	0.016351	UP
Faecalibacterium prausnitzii_G^*^	4.505802	3.075112	−1.162193	0.018581	DOWN
CAG-245 sp000435175^*^	−1.466166	−2.115085	−0.803474	0.023439	DOWN
Anaeroglobus micronuciformis^*^	−5.690037	−3.004993	2.456315	0.030441	UP
Megasphaera sp000417505^*^	−1.164886	−0.571262	0.651322	0.031378	UP
Tidjanibacter inops^*^	−3.764775	−4.847396	−1.533985	0.039835	DOWN
Lachnospira sp000436535^*^	3.040021	1.798285	−1.179473	0.046619	DOWN

The differential microbiota at the species level. Statistical significance was defined as a *p* < 0.05;

* *p* < 0.05;

** *p* < 0.01;

*** p < 0.001.

### The 13 Differential Species Could Distinguish the Active and Remission UC

**Figure 4:**
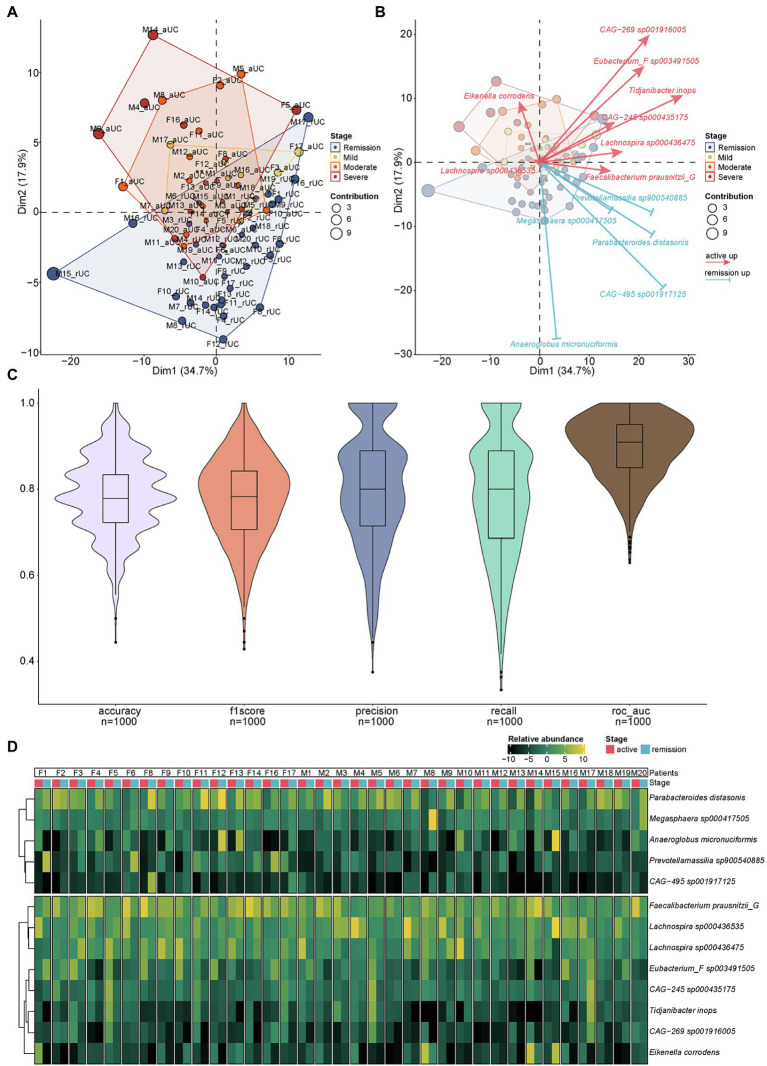
The PCA analysis of different microbiota and the relative abundance of different microbiota for each sample. **(A)** Communities cluster using Principal Component Analysis (PCA). Dim1 and Dim2 are plotted on x- and y-axes. Each point corresponds to each sample. The percentage of variation analyzed by the plotted principal components is shown on the axes. Blue, yellow, orange, and red plots indicate the samples in different disease severity of UC, remission, mild, moderate, and severe, respectively. **(B)** The PCA biplot showed the 13 differential species and their contribution to separation of UC samples. Blue and red arrows indicate the upregulated species in the remission and active stage of UC, respectively. The direction and length of the arrow indicate the difference and the contribution of identification ability of different stages by 13 species above. **(C)** The results of 1,000 prediction simulation. The average accuracy, f1score, precision, recall, and ROC-AUC are 0.79, 0.78, 0.80, 0.78, and 0.90, respectively. **(D)** The heatmap showed the relative abundance of different microbiota at the species level for each sample.

We identified 13 differential species at the species level by paired *t*-test above. Here, we further examined whether the 13 differential species can distinguish different disease severity from active to remission stages including remission, mild, moderate and severe of UC by using the principal component analysis (PCA). The result showed that the 13 differential species can distinguish the different disease severity of UC effectively. The higher the severity of UC, the stronger the ability to distinguish by using these 13 differential species. Meanwhile, the samples from active (mild/moderate/severe) and remission stages of UC patients were roughly distributed in different areas (Figure 4A). The identification direction of different stages by these 13 species was consistent with the distribution of remission stage (below) and active stage (above) of UC, but the identification ability had obvious differences in each species. *Eikenella corrodens* played an important role in distinguishing severe from other disease severity (Figure 4B). We used 1,000 prediction simulations with 75% of the data set as training and 25% as testing to evaluate the performance of this biomarker panel. Then the performance metrics of prediction were calculated. The average accuracy, f1score, precision, recall, and ROC-AUC are 0.79, 0.78, 0.80, 0.78, and 0.90, respectively (Figure 4C). The result (ROC-AUC = 0.90) suggests that the 13 species together have a relatively good ability to predict samples from the active or remission UC. In addition, we checked the abundance of 13 species for each patient (Figure 4D). For each UC patient, the abundance in the active and remission stages is clearly different. The relative abundance of different microbiota can also separate different stages at the levels of phylum, class, order, family, and genus roughly (Supplementary Figure 4). These 13 species together may serve as a biomarker panel contributing to identify the active and remission stages of UC.

### Differential Microbiota Performs Different Molecular Functions and Involves Different Biological Processes

**Figure 5:**
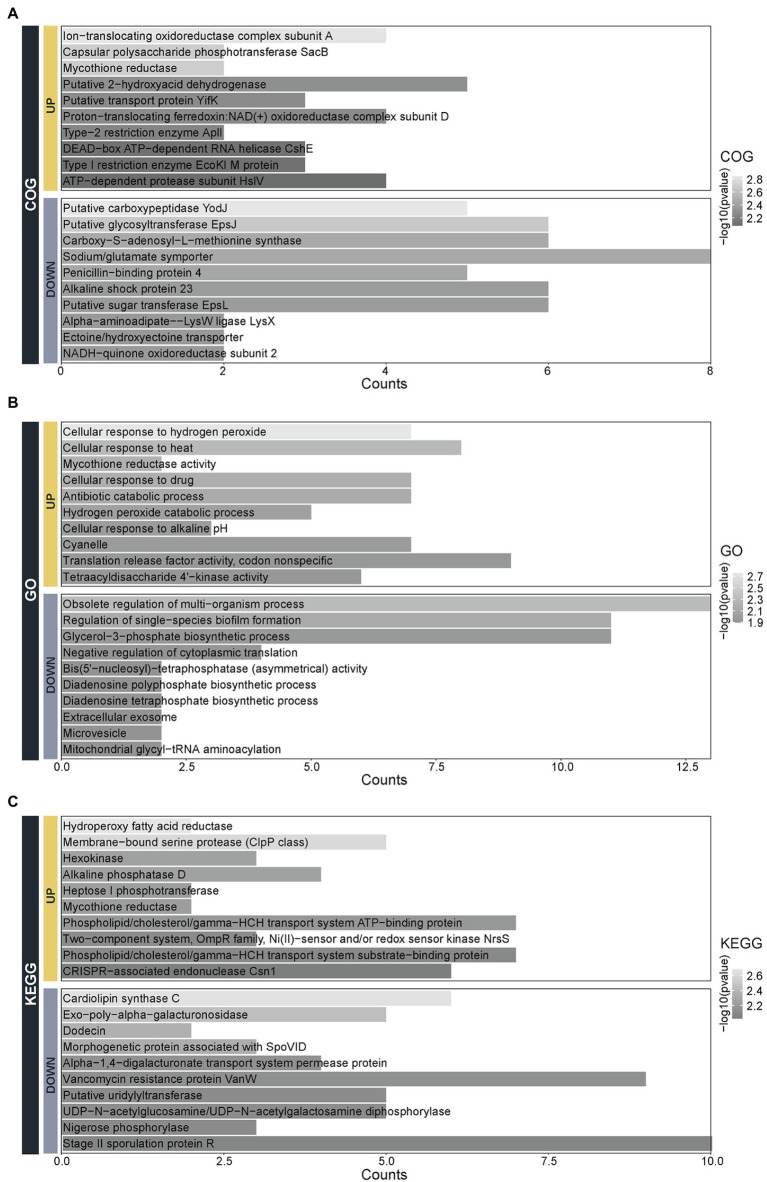
The functional annotation of the 13 species with differential abundance. The function classification of the 13 upregulated and downregulated species by the **(A)** Clusters of Orthologous Groups of proteins (COG), **(B)** Gene Ontology (GO), and **(C)** Kyoto Encyclopedia of Genes and Genomes (KEGG).

To understand the function of 13 differential species, the Clusters of Orthologous Groups of proteins (COG), Gene Ontology (GO), and Kyoto Encyclopedia of Genes and Genomes (KEGG) functional annotations were performed (Figure 5). The molecular functions of the upregulated species are enriched in the Ion-translocating oxidoreductase complex subunit A, cpolysaccharide phosphotransferase SacB, mycothione reductase mainly and so on. While molecular functions of the downregulated species are related to the Putative carboxypeptidase YodJ, putative glycosyltransferase EpsJ, carboxy-S-adenosyl-L-methionine synthase, and so on (Figure 5A). The biological processes of the upregulated species are involved in the processes of cellular response to hydrogen peroxide and heat, the activity of mycothione reductase, hydrogen peroxide catabolic process and so on. In contrast, the biological processes of the downregulated species are enriched in the obsolete regulation of multi-organism, regulation of single-species biofilm formation, glycerol-3-phosphate biosynthetic process, and so on (Figure 5B). The pathways of the upregulated species are mainly involved in the hydroperoxy fatty acid reductase, membrane-bound serine protease (ClpP class), hexokinase, and so on. In contrast, the pathways of the downregulated species are enriched in terms of the cardiolipin synthase C, exo-poly-alpha-galacturonosidase, dodicin, and so on (Figure 5C).

## Discussion

In this study, the paired fecal samples from the same UC patients in the active and remission stages were collected for metagenomics analysis. The time interval between remission and active sample collection was approximately 3 months. The collection interval was relatively short, so the living environment of the patients did not change much, and they all had their own personalized diet, which can exclude the influences of genetic differences, environmental and dietary factors on the intestinal microbiota. The identified intestinal microbiota can be divided into 11 phyla. We found that Bacteroidota, Firmicutes, Proteobacteria, and Actinobacteria were the most widely distributed phyla in UC patients. Consistent with our findings, the previous studies have also found that the dominant intestinal microbiota of UC patients comprise Bacteroidota and Firmicutes primarily (Andersson et al., 2008; Nam et al., 2011; Wills et al., 2014). Inducing epithelial cells to increase anaerobic glycolysis and oxidation may induce a community shift from aerobic bacteria to facultative anaerobes, such as an increase in Proteobacteria (Ni et al., 2017; Gonçalves et al., 2018). It was reported that Enterobacteriaceae under the Proteobacteria phylum increased in IBD patients (Seksik et al., 2003; Baumgart et al., 2007). These suggest that the increase of Proteobacteria may indicate the active stage of UC. Some studies reported that the increase of Proteobacteria and the decrease of Firmicutes in IBD patients were associated with the severity of the disease (Zhou et al., 2018). Our study revealed that Proteobacteria had no difference between the active and remission stages, while the abundance of Firmicutes was increased in the remission stage. It suggests that there is a correlation between Firmicutes and the remission of UC patients.

In our study, the clinical parameters related to disease activity including C-reactive protein (CRP), erythrocyte sedimentation rate (ESR), and hemoglobin (Hb) were tested in the active stage of UC. However, these indicators were not tested in the remission stage because the blood samples of remission were not collected. We have performed the correlation analysis between the abundance of all genus and the clinical parameters of CRP, ESR, and Hb in the active stage. The top 20 genus correlated with Hb, ESR, and CRP were shown in the Supplementary Figure 5. We found that the abundance of genus *Prevotella*, *Enterocloster*, and *Clostridium_P* were positively correlated with Hb, ESR, and CRP, while the abundance of genus *Enterocloster*, *Ruminococcus_D*, and *Parasutterella* were negatively correlated with Hb, ESR, and CRP, respectively.

It was reported that the diversity of intestinal microbiota in UC patients was significantly lower than that in healthy people (Lopetuso et al., 2018; Alam et al., 2020). However, our study showed there was no significant difference in the diversity of intestinal microbiota between the active and remission stages of UC. It suggests that the intestinal microbiota has not changed dramatically between the active and remission stages. Both in active and remission stages, the intestinal microbial diversity of UC patients is different from that of healthy people. Furthermore, our study found the relative abundance of different microbiota can separate the different stages of UC roughly at the levels of phylum, class, order, family, genus, especially at the species level. The species of *Faecalibacterium prausnitzii G*, *CAG-269 sp001916005*, *CAG-245 sp000435175*, *Lachnospira sp000436475*, *Lachnospira sp000436535*, *Eubacterium F sp003491505*, *E. corrodens*, and *Tidjanibacter inops* were upregulated in the active stage of UC, while the species of *Megasphaera sp000417505*, *Anaeroglobus micronuciformis*, *CAG-495 sp001917125*, *Prevotellamassilia sp900540885*, and *Parabacteroides distasonis* were upregulated in the remission. These 13 species could distinguish the active and remission UC effectively, and even roughly distinguish the disease severity (mild/ moderate/severe UC) without the influence of age, gender and so on. *E. corrodens* (belonging to Gammaproteobacteria class) played an important role in distinguishing the severe from other disease severity. Previous studies found that the abundance of Gammaproteobacteria was different in the mild/moderate UC compared with severe UC patients and healthy people (Kedia et al., 2021). The hyperlactate production is related to the intestinal homeostasis disorders of IBD (Hashizume et al., 2003). *Megasphaera elsdenii*, one of the major acid-utilizing bacteria in the intestine, can convert lactate to butyrate. It suggests that the upregulation of *Megasphaera sp000417505* is associated with the remission of UC patients. Previous studies showed *Prevotellamassilia* and *Eubacterium* may be related to the occurrence of constipation-related diseases (Choi et al., 2021). Our study showed the downregulation of *Prevotellamassilia* and upregulation of *Eubacterium* in the remission stage. *P. distasonis*, commonly colonized in the gastrointestinal tract of humans and animals, the abundance of *P. distasonis* has been negatively correlated with many chronic inflammatory diseases including IBD (Ezeji et al., 2021). Some strains of *P. distasonis* were highly effective in alleviating intestinal inflammation and restoring the gut barrier in a mouse model of 2,4,6-trinitrobenzene sulfonic acid (TNBS)- induced colitis (Cuffaro et al., 2020). Our study found *P. distasonis* was upregulated in the remission, which highlighted its potential importance in the homeostasis of gut microbiota.

Through functional annotation, we found the function of upregulated species was enriched in the processes of hydrogen peroxide catabolic process and response to external stimuli such as heat, drug, and pH. Besides, *P. distasonis* possesses catalase and oxidative stress tolerance genes such as KatE gene and the OxyR gene (Ezeji et al., 2021). Oxidative stress is associated with the initiation and progress of IBD while catalase is an important factor in oxidative stress tolerance (Knaus et al., 2017; Liu et al., 2021). While DNA transcription, protein translation, biosynthesis, and spore-forming are accounted for a large proportion of functional annotation in the downregulated species at the remission stage. Studies revealed that spores had an important effect on the disease chronicity due to the reduced susceptibility to biocides (Schaefer et al., 2016). Microbial communities composed of spore-forming bacteria are resistant to antimicrobial therapies particularly (Ðapa et al., 2013; Paredes-Sabja et al., 2014). Our study showed the sporulation function was downregulated in the remission stage of UC, which supports that sporulation is associated with long-term maintenance of remission.

In summary, our study found 13 species with differential abundance from the paired fecal samples of the same patients, which could effectively distinguish the active and remission stages of UC. The 13 species together may serve as a biomarker panel contributing to identify the active and remission stages of UC. The limitations of the current study are the sample size of UC patients is relatively small, the healthy people are not included as healthy controls, which will be enrolled and increase the sample size in the future study. This study provides a valuable clinical reference for the treatment of UC patients by FMT or other therapeutic methods.

## Data Availability Statement

The raw sequence data reported in this paper have been deposited in the Genome Sequence Archive in National Genomics Data Center, Chinese Academy of Sciences, under accession number HRA001567 that is publicly accessible at https://bigd.big.ac.cn/gsa-human/browse/HRA001567. Further information and requests for analysis code should be directed to and will be fulfilled by the corresponding author, Danfeng Lan (ang778@live.cn).

## Ethics Statement

The studies involving human participants were reviewed and approved by the Institutional Review Board for Clinical Research of the First Affiliated Hospital of Kunming Medical University. Written informed consent to participate in this study was provided by the participants’ legal guardian/next of kin. Written informed consent was obtained from the individual(s), and minor(s)’ legal guardian/next of kin, for the publication of any potentially identifiable images or data included in this article.

## Author Contributions

DL, SD, and YC constructed the concept of the whole study. RZ performed bioinformatic analyses. RZ, JT, and CX analyzed the results and wrote the manuscript. QN, GL, JL, JZ, and YM collected clinical samples. DL and SD revised the manuscript. All authors contributed to the article and approved the submitted version.

## Funding

This work was supported by the National Natural Science Foundation of China (81860105, 82170550, and 32160153); the Applied Basic Research Projects of Yunnan Province (2019FE001-140, 2019FB050, and 202105AD160008); and the Yunnan Health Training Project of High Level Talents (H-2018041 and YNWR-QNBJ-2020-236).

## Conflict of Interest

The authors declare that the research was conducted in the absence of any commercial or financial relationships that could be construed as a potential conflict of interest.

## Publisher’s Note

All claims expressed in this article are solely those of the authors and do not necessarily represent those of their affiliated organizations, or those of the publisher, the editors and the reviewers. Any product that may be evaluated in this article, or claim that may be made by its manufacturer, is not guaranteed or endorsed by the publisher.

## Supplementary Material

Supplementary Figure 1 The distribution of clean reads for each sample at the active and remission stages UC. X-axis: each column represented each sample, Y-axis: the number of clean reads. Each point indicated one species.

Supplementary Figure 2 The composition of intestinal microbiota in the active and remission stages of UC. X-axis: each column represent each sample, Y-axis: the abundance of the intestinal microbiota of different phyla in each sample.

Supplementary Figure 3 The LEfSe analysis of intestinal microbiota and the covariate evaluation of the differential microbiota. **(A)** Comparisons of the relative abundance of bacterial found by LEfSe analysis in the following taxa: Negativicutes, FiricutesC, Tannerellaceae, Megaphaeraceae, Neisseriaceae, Parabacterioides, Prevotellamassilia, AM51_8, Anaeroglobus, Eikenella, Eubacterium_F, Blautia, Duodenibacillus, Faecalibacterium. The straight line indicates the group means, and the dotted line indicates the group medians. **(B)** Partial least squares (PLS) analysis (left) and regression coefficient plot (right) of each variate. Each column indicates one variate. The values both left and right Y-axis evaluate the importance of each variate.

Supplementary Figure 4 The heatmap showed the relative abundance of different microbiota. The relative abundance of different microbiota in the phylum, class, order and genus level for each sample.

Supplementary Figure 5 The correlation analysis between the abundance of all genus with the clinical parameters. The correlation analysis was performed between the abundance of all genus with the clinical parameters related to disease activity including Hb **(A)**, ESR **(B)** and CRP **(C)** in the active stage of UC.

Supplementary Table 1 The clinical information and sequencing information for each sample.

Supplementary Table 2 Statistics on clean reads of each species.
