# Supplementary figures and images for: The Distinguishing Bacterial Features From Active and Remission Stages of Ulcerative Colitis Revealed by Paired Fecal Metagenomes
Source: Front Microbiol. 2022 Jun 21;13:883495. doi: 10.3389/fmicb.2022.883495 (PMC9253600; doi:10.3389/fmicb.2022.883495)

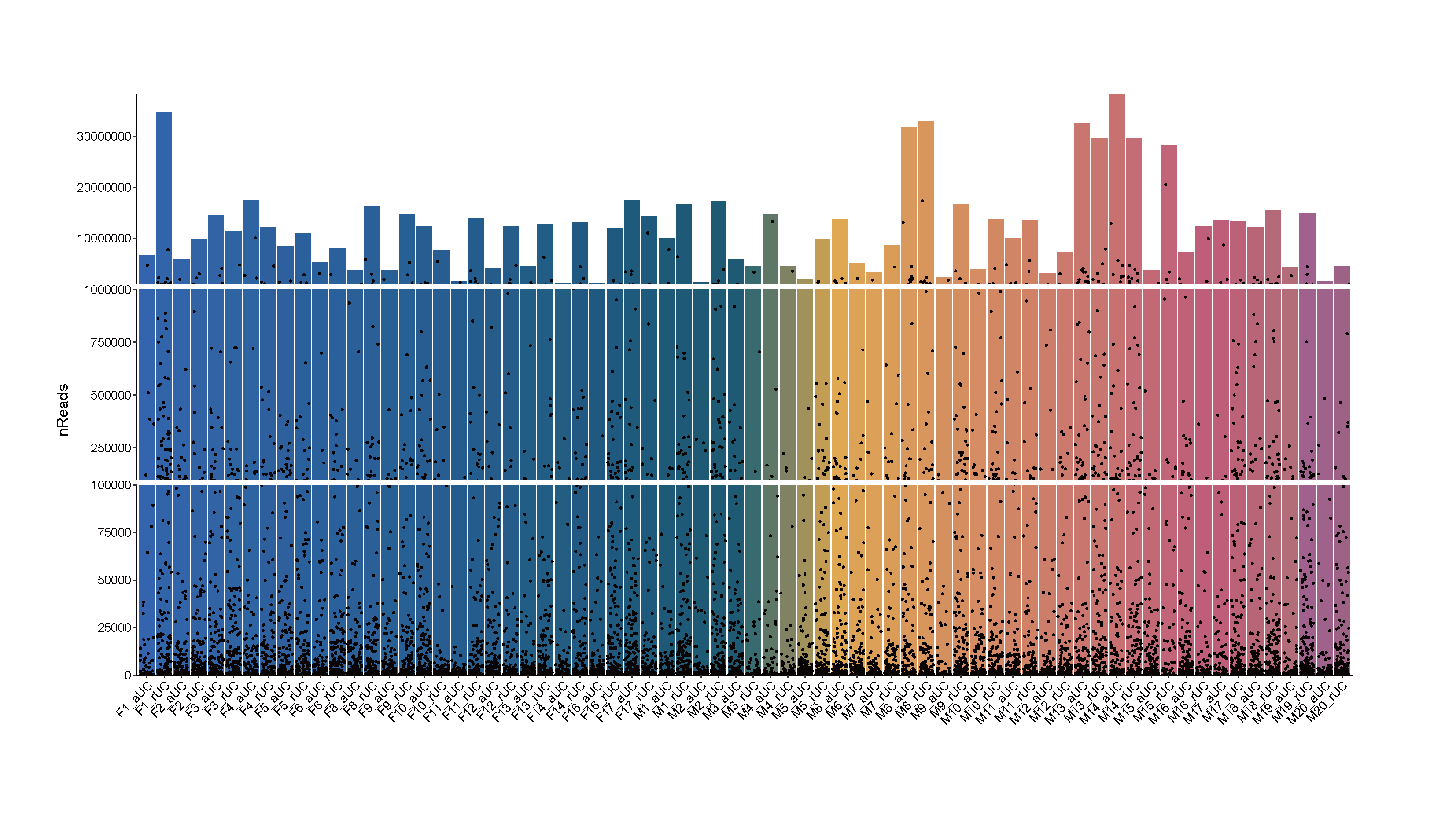

Supplement: Supplementary Figure 1 — The distribution of clean reads for each sample at the active and remission stages UC. X-axis: each column represented each sample, Y-axis: the number of clean reads. Each point indicated one species. [file Image_1.TIF]

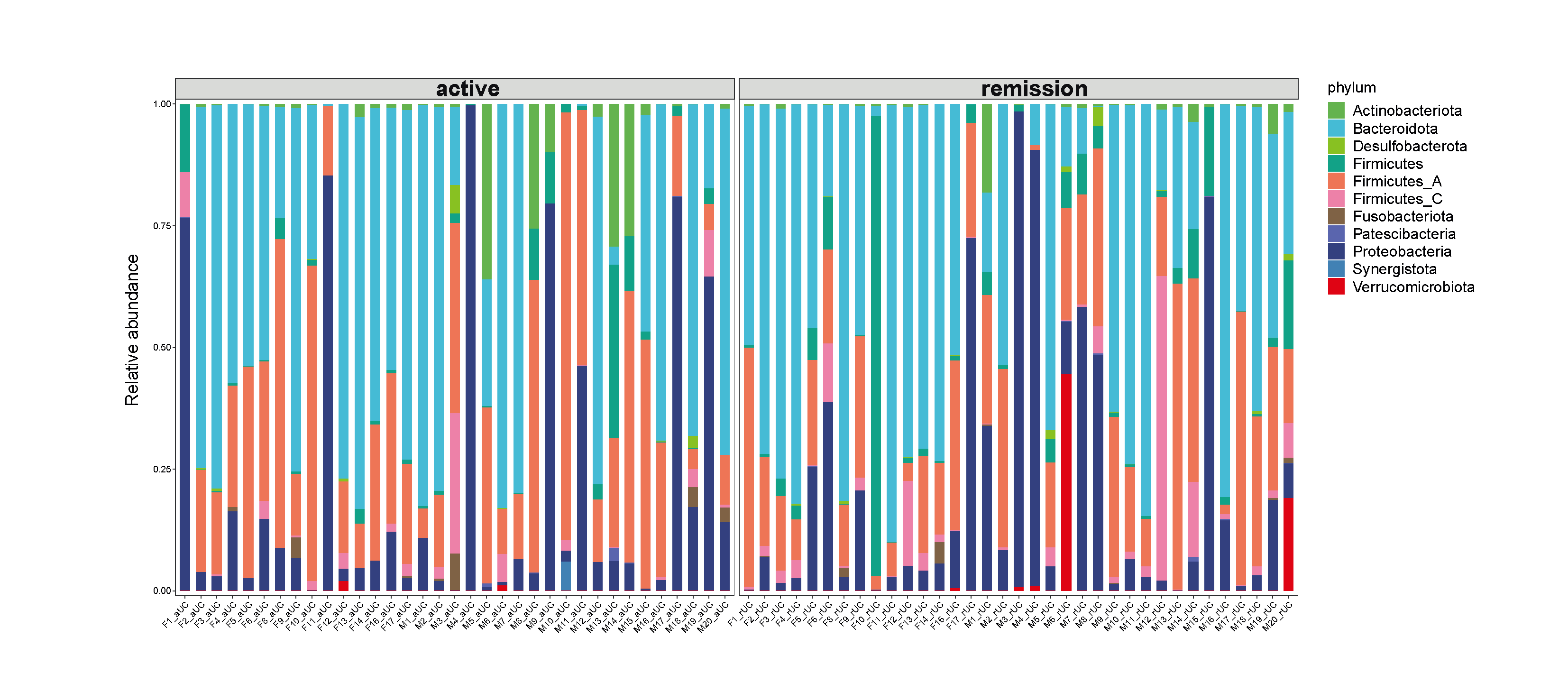

Supplement: Supplementary Figure 2 — The composition of intestinal microbiota in the active and remission stages of UC. X-axis: each column represent each sample, Y-axis: the abundance of the intestinal microbiota of different phyla in each sample. [file Image_2.TIF]

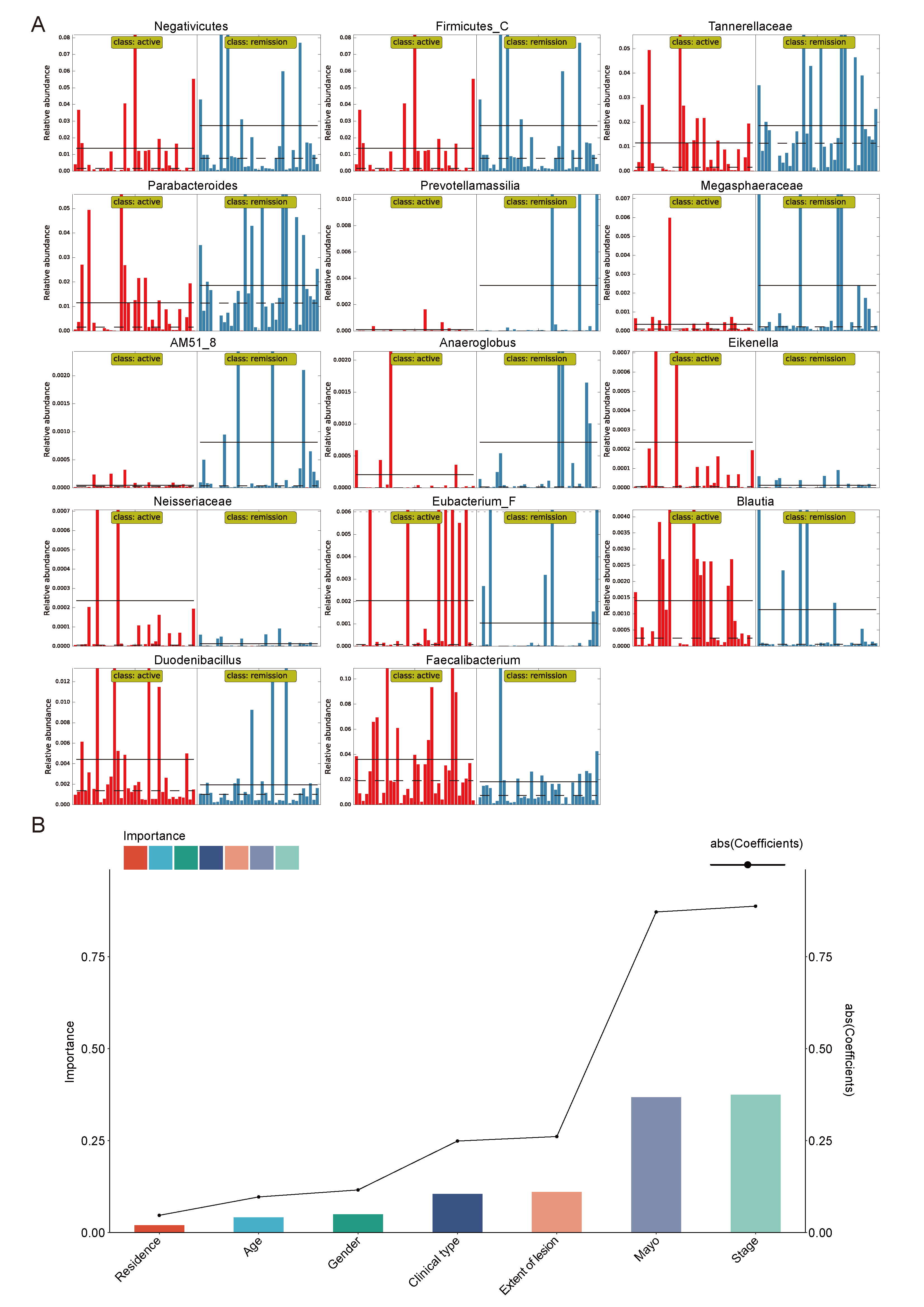

Supplement: Supplementary Figure 3 — The LEfSe analysis of intestinal microbiota and the covariate evaluation of the differential microbiota. (A) Comparisons of the relative abundance of bacterial found by LEfSe analysis in the following taxa: Negativicutes, FiricutesC, Tannerellaceae, Megaphaeraceae, Neisseriaceae, Parabacterioides, Prevotellamassilia, AM51_8, Anaeroglobus, Eikenella, Eubacterium_F, Blautia, Duodenibacillus, Faecalibacterium. The straight line indicates the group means, and the dotted line indicates the group medians. (B) Partial least squares (PLS) analysis (left) and regression coefficient plot (right) of each variate. Each column indicates one variate. The values both left and right Y-axis evaluate the importance of each variate. [file Image_3.TIF]

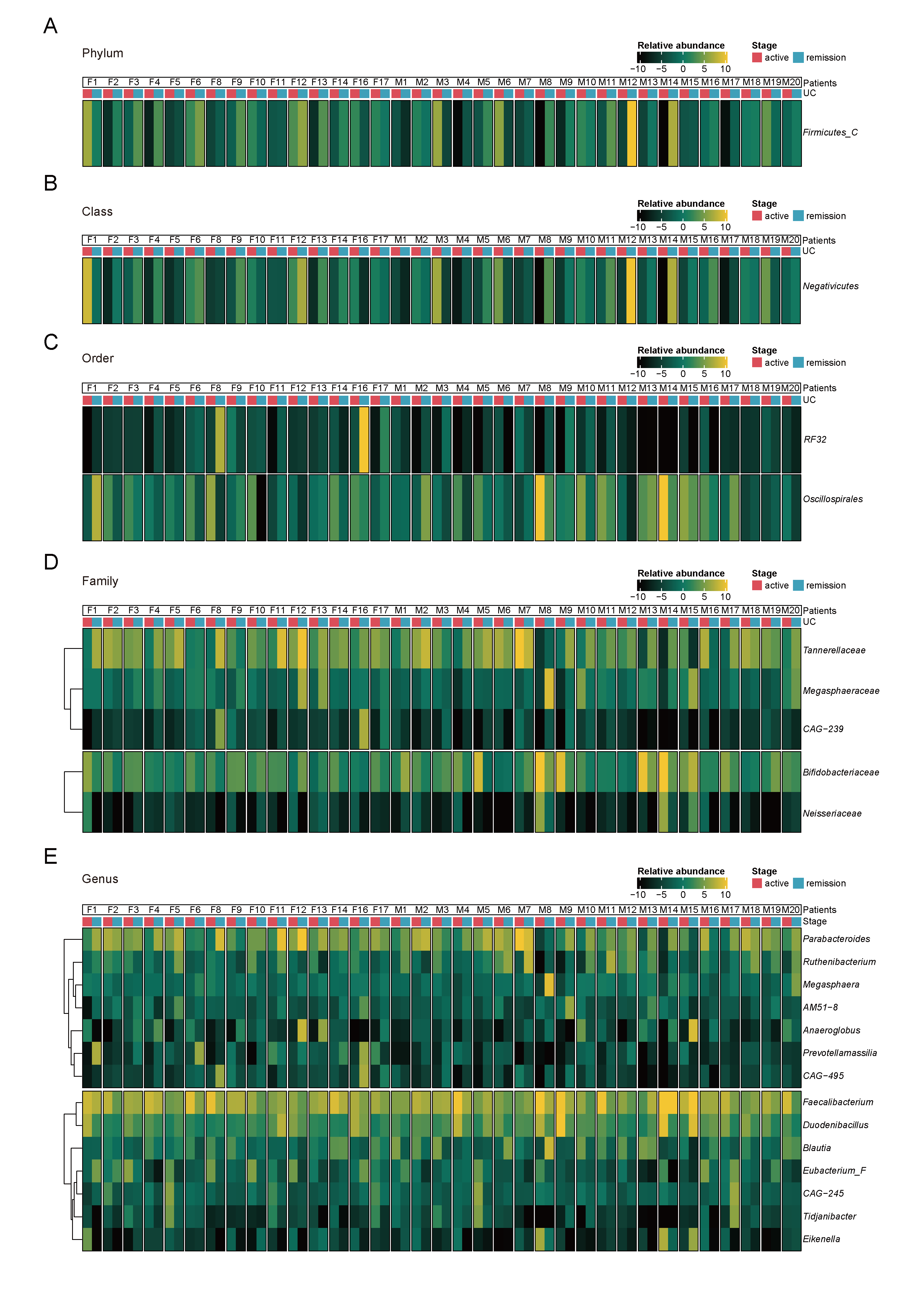

Supplement: Supplementary Figure 4 — The heatmap showed the relative abundance of different microbiota. The relative abundance of different microbiota in the phylum, class, order and genus level for each sample. [file Image_4.TIF]

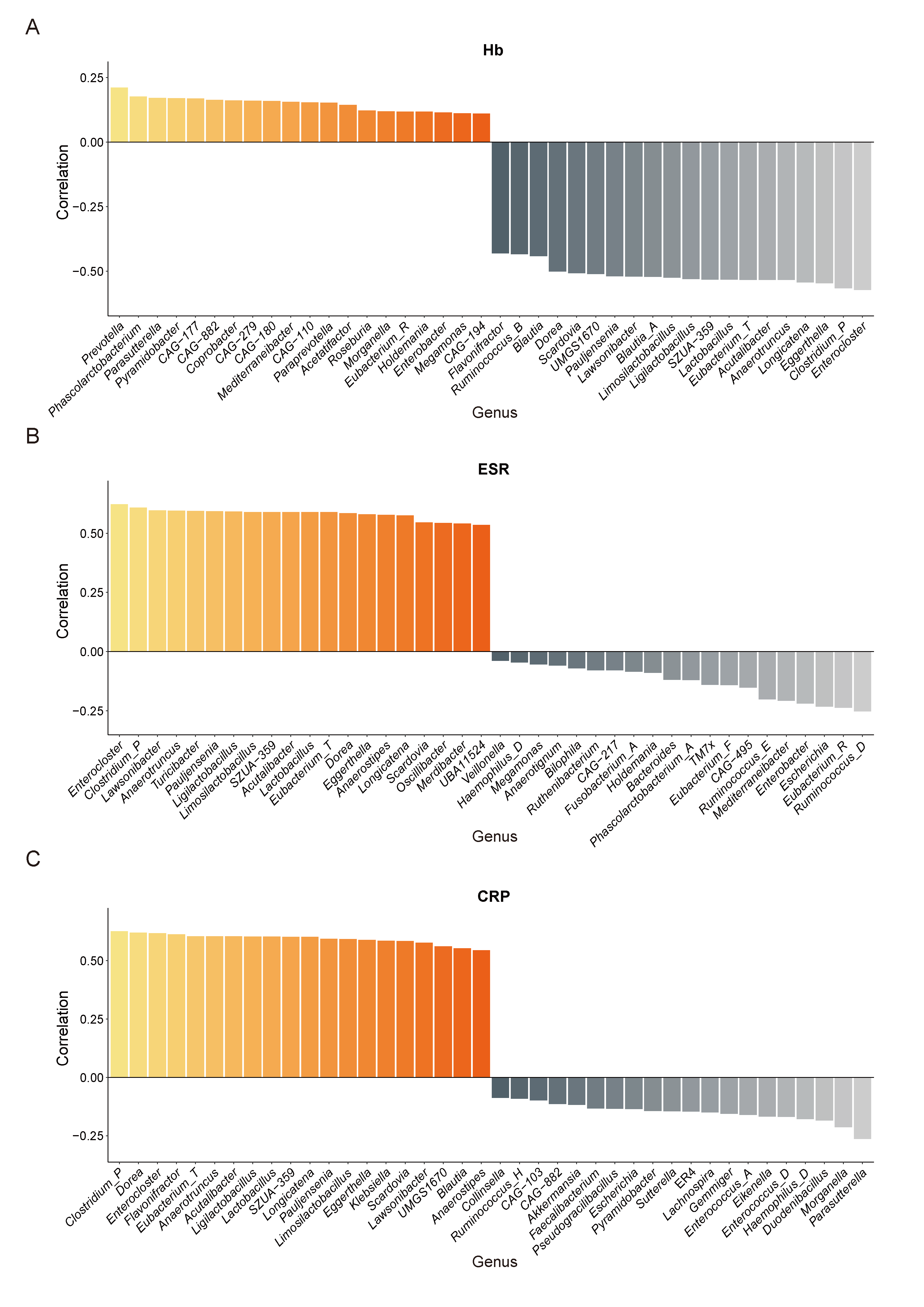

Supplement: Supplementary Figure 5 — The correlation analysis between the abundance of all genus with the clinical parameters. The correlation analysis was performed between the abundance of all genus with the clinical parameters related to disease activity including Hb (A), ESR (B) and CRP (C) in the active stage of UC. [file Image_5.TIF]
